# Absence of low back pain in the general population followed fortnightly over one year with automated text messages

**DOI:** 10.1186/2045-709X-22-1

**Published:** 2014-01-09

**Authors:** Charlotte Leboeuf-Yde, Nadège Lemeunier, Niels Wedderkopp, Per Kjaer

**Affiliations:** 1 Research Department, The Spine Center of Southern Denmark, Hospital Lillebælt and Institute of Regional Health Research, University of Southern Denmark, Østre Hougvej 55, DK5500 Middelfart, Denmark; 2 Complexité, Innovation et Activités Motrices et Sportives, Bâtiment 335, UFR STAPS, Université Paris Sud-11, Orsay Cedex 91405, France; 3 Institut Franco-Européen de Chiropraxie, 72 Chemin de la Flambère, 31300 Toulouse, France; 4 Orthopaedic Department, Center for Spine Surgery, Hospital of Lillebaelt, Institute of Regional Health Service Research and Center for Research in Childhood Health, University of Southern Denmark, Østre Hougvej 55, DK5500 Middelfart, Denmark; 5 Institute of Sports Science and Clinical Biomechanics, University of Southern Denmark, Campusvej 55, DK5230 Odense M, Denmark

**Keywords:** Low back pain, General population, Population study, Episodes, Epidemiology, Text messages, Longitudinal, Prospective, Survey

## Abstract

**Background:**

Over one year, the majority of patients with low back pain (LBP) from the secondary care sector could not report a single week without LBP and few could report a non-episode, defined as at least one month without LBP. Presumably, non-episodes would be more common in the general population. The aim of this study was to assess the usefulness of this definition of ´”non-episodes”, by studying their presence over one year in the general population. Specifically, we wanted to: 1) determine the prevalence of non-episodes, 2) identify the proportion of study participants who could be classified as being in a non-episode at the end of the observation period, and 3) estimate the proportion of participants classified as having at least two separate non-episodes.

**Methods:**

Danes, aged 49/50, who previously participated in a population-based study on LBP received fortnightly automated text (SMS) messages over one year. Each time, participants reported the number of days with LBP in the preceding fortnight. Fortnights with 0 days of LBP were defined as ‘zero-fortnights’ and two such fortnights in a row (one month) were defined as a ‘non-episode’. Estimates are reported as percentages with their 95% confidence intervals in brackets.

**Results:**

Two hundred and ninety-three people were invited to participate. Of these, 16 declined participation and 16 were excluded because they failed to return their text message at least 20 of the 26 times, leaving 261 in the current analyses. Of these, 11% (2-22) never reported a zero-fortnight. In all, 83% (78-88) had at least one non-episode throughout the study period and the proportion of participants classified as being in a non-episode at the end of the study was 59% (53-65). The percentage of individuals with at least two non-episodes was 52% (46-58).

**Conclusions:**

It is possible to differentiate people from the general population as having or not having episodes of LBP using the definition of absence of LBP over one month as the measure. Non-episodes were far more common in the general population than in the secondary care sector, suggesting it to be a potentially useful definition in research.

## Background

Low back pain (LBP) is suspected to be an episodic condition [1]. However, unless an episode can be clearly identified, it would be difficult to study the pattern of LBP. Such identification would depend on a clear demarcation between episodes and non-episodes. Thus two non-episodes would be needed to detect one episode.

In 2002, following a review of the scientific literature, de Vet et al suggested that a ‘non-episode’ of LBP should be defined as a period of at least one month without symptoms [2]. However, at the time, they could find no evidence supporting the relevance of this definition. Nor was there an obvious biological rationale behind this definition.

In the absence of a gold standard and an objective measurement of absence of LBP, it is nevertheless relevant to attempt to validate this definition by comparing its prevalence in various populations. Because the nature and severity of LBP differ between study populations, it would be expected that patients in the primary care sector more commonly report non-episodes and that these non-episodes last longer than for patients in the secondary care sector. Further, it would be expected that the prevalence of non-episodes would be even higher and of longer duration in the general population.

Two studies were previously conducted in a specialized outpatients back hospital on patients aged 18-60 years, with LBP of 2-12 months’ duration. A secondary analysis of data from these two studies revealed that the vast majority of these patients could not report even a single week without LBP during the one-year study period [3]. The proportion of study participants with at least one non-episode during the study period was only approximately 20% and just 5% were classified as being in a non-episode at the time-point of the end of the study.

We had access to automated frequent text message data from a study sample of 49/50-year olds from the general population. This type of data collection is suitable to collect few and short questions at pre-specified time intervals. This made it possible to obtain answers to the following research questions during a one-year period:

1. What was the prevalence of at least one non-episode?

2. What was the proportion of study participants who could be classified to be in a non-episode at the end of the observation period?

3. What was the proportion of participants, who could be classified as having at least two separate non-episodes?

Our aim was to provide further information on the relevance of the definition of being free of LBP for at least one month (‘a non-episode’) by comparing the results obtained in the general population to the results previously obtained in the secondary care sector [3]. Therefore, the first two research questions and the methods of analysis were identical to those in the previous study [3].

## Methods

### Brief summary of the study

Automated text messages were collected every fortnight over one year on a study sample from the general population. Information was sought on the frequency (but not severity) of LBP. This information was used in order to establish the duration of pain-free periods in individuals and to establish the frequency of non-episodes (i.e. periods without pain for at least one month).

### Study subjects

In the year 2000, a randomly selected group of 625 Danes of approximately 40 years of age, living on the island of Funen, Denmark, were invited to participate in a study on LBP, which included an MRI study [4]. The participation rate was 66% and the study subjects were considered representative of the Danish population with only slight deviations from the profile of the general population on schooling and occupational status [5]. At the baseline study, the prevalence estimate of LBP was somewhat higher than could be expected in the general Danish population, and for this reason, it is possible that the original study sample was slightly biased towards those with LBP and those who had an interest in learning more about their problem.

Two consecutive follow-up studies were carried out 4 and 8 years later and, in the year 2009, an invitation to participate in the current study was extended to those 293 people who were still in the study at the time of the third survey. These people were all 50 years of age during the year of that study.

### Fortnightly data collection with text messages

Automated text messages were sent to the participants every fortnight over one year (26 times), using the SMS-Track-Questionnaire [6]. Thus they received two standardized questions relating to their low back, one on pain and the other on sick leave. Only the topic of LBP will be dealt with in this report.

The question on pain was: “Using a number from 0 to 14, please answer how many days your back has bothered/troubled you over the past two weeks”. The answer was provided by returning a text message with a number, e.g. “0” if there had been no days with bothersome LBP during the preceding fortnight or “5” if the fortnight had contained five days with LBP problems. The automated text messages were automatically transferred to a data file. Data were password-protected and were kept at a designated server.

One year earlier in a questionnaire survey, the study subjects had been asked the same question about being “bothered” by their LBP and the question had been accompanied by an anatomical drawing to indicate the lumbar area. Also, the term ‘bothersomeness’ has been used for various medical conditions and was shown by Dunn and Croft in 2005 to be a valid measure of LBP severity, meaning that mainly non-trivial LBP would be reported when using this term [7].

The text message replies went straight into a data file, which could be accessed by the researchers at any time. Participants who failed to respond or who misunderstood how to do this (e.g. by sending ordinary text messages) could thus be contacted as soon as the erroneous answer was available for inspection, making it possible to provide them with clarification and/or assistance, which optimized both the response rate and the quality of data. A research secretary was responsible for this throughout the study period.

### Ethical considerations

Ethics and data management approvals were obtained from the Ethics Committee (ref. number 200000042) for the original study and the Danish Data Protection Agency for the SMS-Track data collection (ref number 2000-53-0037). There are built-in encryptions of the SMS systems in all telecommunication companies in Denmark, which provide protection of participants’ data when data are exchanged between participants and the server.

### User friendliness and validity of text-messaging data

This method of data collection, although relatively new, had been validated in a previous study [8]. Provided clear information is given and there is vigilance in relation to the text message returns, particularly at the onset of the study, it is possible to obtain a high response rate and relevant data [8].

### Data management and analysis of data

Study subjects who did not return their text message at least 20 of the 26 times that they were sent, were excluded from the analysis. The reason for this was that, when inspecting the data file, it was more difficult to understand and relate to the individual reporting pattern when six or more answers (‘cells’) were missing.

A comparison was made between responders and non-responders, defining ‘responders’ as those who participated in the current study and whose data were used for analyses, and defining ‘non-responders’ as those who failed to participate and therefore whose data were excluded from the analyses. The variables used for this purpose had been collected at baseline, when the participants were approximately 40 years of age, in a questionnaire containing some descriptive information.

In relation to the text-message data, fortnights with 0 days of LBP were defined as ‘zero-fortnights’ and two zero fortnights in a row were defined as a ‘non-episode’, following de Vet et al’s definition of one month without symptoms [2]. Missing values were filled in through manual imputation by dividing the individual’s total number of days with LBP by the total number of SMS-messages. Also computerized imputation was performed, using the software package STATA [9] and the *mi* command. This method included other psycho-social and demographic variables available in the data set. The two methods gave very similar results. However, the manual method was preferred because visual inspection indicated that, occasionally, some of the imputed data stood out as being improbable. This decision was based on the observation that data reporting was highly consistent throughout the study. Most participants reported LBP similarly throughout the study; either as no LBP, few days of LBP, or many days of LBP.

A manual analysis was undertaken. First, the maximum number of zero-fortnights in a row was identified for each individual. Second, the number of participants who were classified as having at least one non-episode was counted, as was the number of those who were in a non-episode at the time of the last week of the study. Finally, the number of participants with at least two non-episodes was identified. The analyses were undertaken twice, blind to the previous results, with no differences in findings. Estimates are reported with their 95% confidence intervals in brackets. As in the previous study [3], no analyses were undertaken for subgroups or potential modifiers.

## Results

### Sample size, response rate, comparison between responders and non-responders, and missing cells

In all, 293 people were invited to participate. Of these, 16 declined participation and 16 were excluded because they did not return their SMS messages at least 20 times, leaving 261 (89%) participants in the current analyses, of whom 142 were women and 119 men. The comparison of responders and non-responders shows men, people with only basic schooling, and those who had more than 30 days of LBP at baseline to be somewhat over-represented among the non-responders (Table 1). Conversely, those who were employed were somewhat over-represented among the responders (Table 1). The occasional text message response was missing for 76 of the participants. Thus, imputation was carried out in 2% of the cells (129/6786).

**Table 1:** Description of responders and non-responders in a study sample aged 50 years from the general Danish population (N = 261)

**Information obtained at base-line**	**Responders (n = 261) %**	**Non responders (n = 16) and persons excluded from the analyses (n = 16) %**
**Gender**		
Male	46	53
Female	54	47
**Employment status**		
Self employed	7	7
Assisting spouse	0	1
Employed	88	76
Unemployed	3	7
Pensioners	1	6
Others outside labour force	2	3
**Highest educational level**		
Basic school	19	28
General upper-secondary education	2	2
Vocational education and training		
Short-cycle higher education	33	27
Medium-cycle higher education	21	19
Long-cycle higher education	19	18
	5	6
**Number of days with LBP past year**		
0 days	33	25
1-30 days	46	44
>30 days	21	32

Variables used for comparison were obtained through a questionnaire at baseline, when the study subjects were 40 years old.

### The maximum numbers in a row of fortnights without LBP

**Figure 1:**
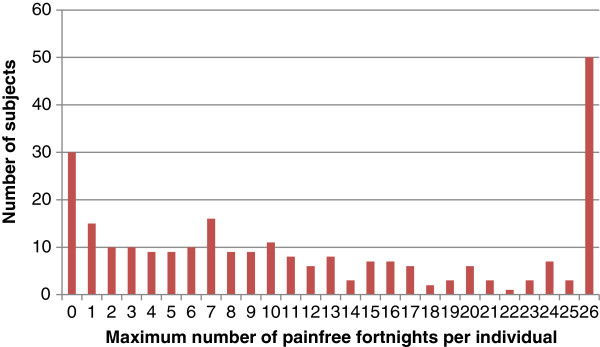
The maximum number of pain-free fortnights in a row over one year with results from a study sample of 261 Danes aged 50 years, who participated in a study on low back pain, in which data were obtained with fortnightly text messages (SMS-Track).

The most commonly reported numbers of zero fortnights over one year were found at the two extremes; none or all. In all, 11% (12-22) failed to report a zero-fortnight, whereas 19% (14-24) did not report a single day of LBP throughout the entire study. The data were distributed fairly evenly between these two. See Figure 1 for details.

### The prevalence of non-episodes throughout the one-year period and at the end of the observation period

At least one non-episode throughout the study period was classified in the text responses of 83% (78-88) participants and the proportion of participants classified as being in a non-episode at the end of the study was 59% (53-65).

### The percentage of participants classified as having at least two separate non-episodes

During the study period, the proportion of participants who had at least two separate non-episodes was 52% (46-58).

## Discussion

### Summary of findings and comparison to a previous similar study

This is the second study in which SMS-Track data for non-episodes were studied for a specific study population over one year. The first study revealed non-episodes to be rare in patients with LBP who had been referred to a specialized back hospital [3]. According to the results in this second study, however, people in the general population enjoy long periods of non-episodes and about 1/5th do not appear to have experienced any LBP at all. Our results therefore indicate that this definition of non-episodes is potentially useful, as its prevalence is logically different in these two very different study populations. In addition, we found that there were a fairly large number of people with at least two non-episodes, indicating that there would be some episodes in this population, not only people either with or without LBP.

In LBP research, using ordinary questionnaire data, it is customary to establish LBP status at the end of the study period, such as at a 6-month or a 12-month follow-up. If ‘recovery’ were studied after twelve months in patients with LBP attending the secondary care sector, it is likely that only a small minority would be considered to be well [3] whereas in the general population, not surprisingly, somewhat more than half would be considered LBP-free.

### Strengths and limitations of the study

A strength of this study is that it deals with a randomly selected sample from the general population. Another strength is that the study participants were all of the same age, which means that there would be no modifying effect of age. This narrow age bracket means, though, that results may not be transferable to those much younger or older. The frequent data collection and the medium through which it was conducted seemed acceptable to the participants, as there were only 16 people who produced insufficient data for inclusion in the study and among the participants only 2% of all cells were missing.

Although the response rate was apparently high, 277 out of 293 (94%), the final study sample did, in fact, only represent 277/412 (67%) of the baseline study sample and just 277/625 (44%) of those initially targeted. This probably results in a highly selected study sample, but if and how this could have affected our results is not known. This declining participation rate in studies with multiple follow-ups is a known phenomenon but it is often ignored in studies with several follow-ups, as response rates are commonly calculated on the basis of those invited in the immediately preceding survey [10]. Our response rate therefore does not compare unfavourably to those of many other similar studies. However, the purpose of this study was not to establish exact prevalence estimates of episodes and non-episodes but rather to investigate the usefulness of the definition of ‘non-episode’. Other age groups or sub-samples of the general population could, of course, have different profiles.

Another positive aspect of the current study is that the frequent data collection and short reference period is likely to minimise recall inaccuracies. Also, as the data reporting was very consistent (those without pain kept on reporting no pain and those with pain reported pain in a similar manner throughout the whole study period), it is unlikely that the imputed values (only 2% of all cells which were spread over several people) would have falsified the final results.

Nevertheless, when comparing non-episode data between studies, it would be important to take into account the recall period. In this study, we used two weeks. This meant that a non-episode would depend on the absence of LBP over two consecutive fortnights (i.e. one month). However, if one pain-free fortnight was found next to one pain-free week on each side, this would not be identified, if each of those neighbouring fortnights contained at least one day with LBP, as it cannot be seen, in which of the two weeks this day is located. If data had been collected weekly, more pain-free months could therefore have been identified. It is therefore possible that there is some under-reporting of pain-free months in the current study. The choice to collect data fortnightly only was made in order not to fatigue our participants with too many text-messages over the study period. We assumed that it would be easier to obtain compliance 26 rather than 52 times over one year, particularly as this study was conducted on the general population.

Although this study was able to provide unique course data over one year, no information is available on the fluctuation in symptom severity. This would have necessitated more complex questions and answers which were unsuitable for this type of data collection. However, when studying the absence of LBP, symptom severity does not appear important. If a person reports having pain, regardless of its intensity, it is likely that classifying them as not being absent of pain is the correct choice.

## Conclusions

Contrary to patients in the secondary care sector, the vast majority of 50-year old individuals from the general population would meet de Vet et al’s definition from 2002 [2] of a ‘non-episode’. This makes it possible to use one month of absence of LBP as a measure of demarcation between episodes and non-episodes in the general population, and as pointed out by de Vet et al, this seems to be a realistic option in view of the brief recall period [2]. However, whether this definition is biologically relevant, is a different question altogether.

## Competing interests

The authors declare that they have no competing interests.

## Authors’ contributions

PK was responsible for the epidemiologic study that formed the basis for this work. NW brought expertise on the use of the SMS-Track system. CLY instigated the current study, performed the analysis and wrote the first draft. All the authors were involved in discussing the concepts, writing the manuscript and have approved the final version.
